# 2-(4-Chloro­phen­yl)-3,5-dimethyl-1λ^6^,2-thia­zine-1,1-dione

**DOI:** 10.1107/S1600536811038852

**Published:** 2011-09-30

**Authors:** Rostam R. Braim, Kamal Aziz Ketuly, A. Hamid A. Hadi, Hamid Khaledi

**Affiliations:** a Department of Chemistry,University of Salahaddin-Erbil, KurdistanIraq; b Department of Chemistry, University of Malaya, 50603 Kuala Lumpur, Malaysia

## Abstract

In the title compound, C_12_H_12_ClNO_2_S, the S atom is displaced by 0.708 (2) Å out of the plane through the remaining atoms of the thia­zine ring (r.m.s. deviation = 0.0823 Å). This plane makes a dihedral angle of 89.33 (7)° with the phenyl ring. In the crystal, adjacent mol­ecules are connected through C—H⋯O hydrogen bonds into layers parallel to the *bc* plane.

## Related literature

For the structure of the 4-meth­oxy­phenyl analogue, see: Fanghanel *et al.* (1998 ▶). For some reactions of sultones and sultams, see: Imam Ismail (1990 ▶).
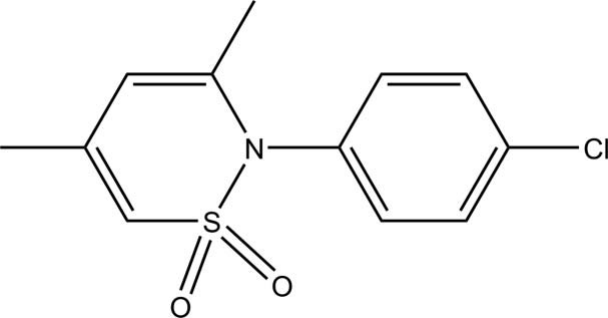


## Experimental

### 

#### Crystal data

C_12_H_12_ClNO_2_S
*M* *_r_* = 269.74
Monoclinic, 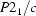

*a* = 11.2237 (1) Å
*b* = 15.1606 (2) Å
*c* = 7.8752 (1) Å
β = 108.4503 (8)°
*V* = 1271.15 (3) Å^3^
*Z* = 4
Mo *K*α radiation
μ = 0.45 mm^−1^
*T* = 100 K
0.28 × 0.24 × 0.19 mm

#### Data collection

Bruker APEXII CCD diffractometer
Absorption correction: multi-scan (*SADABS*; Sheldrick, 1996 ▶) *T*_min_ = 0.884, *T*_max_ = 0.919
11365 measured reflections
2768 independent reflections
2447 reflections with *I* > 2σ(*I*)
*R*_int_ = 0.027

#### Refinement

*R*[*F* ^2^ > 2σ(*F* ^2^)] = 0.030
*wR*(*F* ^2^) = 0.080
*S* = 1.04
2768 reflections
156 parameters
H-atom parameters constrained
Δρ_max_ = 0.38 e Å^−3^
Δρ_min_ = −0.43 e Å^−3^

### 

Data collection: *APEX2* (Bruker, 2007 ▶); cell refinement: *SAINT* (Bruker, 2007 ▶); data reduction: *SAINT*; program(s) used to solve structure: *SHELXS97* (Sheldrick, 2008 ▶); program(s) used to refine structure: *SHELXL97* (Sheldrick, 2008 ▶); molecular graphics: *X-SEED* (Barbour, 2001 ▶); software used to prepare material for publication: *SHELXL97* and *publCIF* (Westrip, 2010 ▶).

## Supplementary Material

Crystal structure: contains datablock(s) I, global. DOI: 10.1107/S1600536811038852/vm2124sup1.cif

Structure factors: contains datablock(s) I. DOI: 10.1107/S1600536811038852/vm2124Isup2.hkl

Supplementary material file. DOI: 10.1107/S1600536811038852/vm2124Isup3.cml

Additional supplementary materials:  crystallographic information; 3D view; checkCIF report

## Figures and Tables

**Table 1:** Hydrogen-bond geometry (Å, °)

*D*—H⋯*A*	*D*—H	H⋯*A*	*D*⋯*A*	*D*—H⋯*A*
C6—H6⋯O1^i^	0.95	2.57	3.3384 (18)	138
C9—H9⋯O2^ii^	0.95	2.44	3.3339 (19)	156

Symmetry codes: (i) 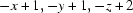; (ii) 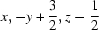.

**Fig. 1.**
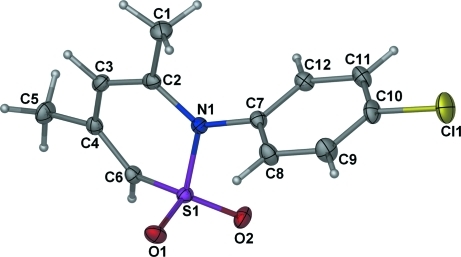
Molecular structure of the title compound with thermal ellipsoids at the 50% probability level. Hydrogen atoms are drawn as spheres of arbitrary radius.

## supplementary crystallographic information

### Comment

Sultones are cyclic sulfonate esters of hydroxy sulfonic acids and may be saturated or unsaturated. It is well known that the reaction of unsaturated sultones with primary amines forms the corresponding sultams, a cyclic sulfonamide in which the S—N bond is part of the ring (Imam Ismail, 1990). The title sultam compound was obtained through the reaction of the sultone, 4,6-dimethyl-2,2-dioxo-1,2-oxathiine, with *p*-chloroaniline. Similar to what was observed in the structure of the 4-methoxyphenyl analogue (Fanghanel *et al.*, 1998), the thiazine ring adopts a half-chair conformation with the S atom displaced by 0.708 (2) Å from the plane passing through the remaining five atoms of the ring, C2,C3,C4,C6 and N1. This plane and the phenyl ring make a dihedral angle of 89.33 (7)°. In the crystal, intermolecular C—H···O hydrogen bonds connect the molecules to form a two-dimensional network parallel to the *bc* plane (Table 1).

### Experimental

A mixture of 4,6-dimethyl-2,2-dioxo-1,2-oxathiine (1.6 g, 0.01 mol) and *p*-chloroaniline (1.28 g, 0.01 mol) in a conical flask was heated at 150°C for an hour. The content was cooled to room temperature and then washed with an aqueous solution (10 ml) of HCl (0.1 N). The white solid was collected, washed with water and dried over silica-gel. The X-ray quality crystals were obtained from a methanol solution at room temperature.

### Refinement

Hydrogen atoms were placed at calculated positions at distances H—C*_sp2_* = 0.95 Å and *H*—*C**_methyl_**=* 0.98 Å and were treated as riding on their parent atoms, with *U*iso(H) = 1.2 (1.5 for methyl)*U*eq(C).

### Crystal data

**Table:**

C_12_H_12_ClNO_2_S	*F*(000) = 560
*M**_r_* = 269.74	*D*_x_ = 1.409 Mg m^−^^3^
Monoclinic, *P*2_1_/*c*	Mo *K*α radiation, λ = 0.71073 Å
Hall symbol: -P 2ybc	Cell parameters from 4756 reflections
*a* = 11.2237 (1) Å	θ = 2.7–30.5°
*b* = 15.1606 (2) Å	µ = 0.45 mm^−^^1^
*c* = 7.8752 (1) Å	*T* = 100 K
β = 108.4503 (8)°	Block, colorless
*V* = 1271.15 (3) Å^3^	0.28 × 0.24 × 0.19 mm
*Z* = 4	

### Data collection

**Table:**

Bruker APEXII CCD diffractometer	2768 independent reflections
Radiation source: fine-focus sealed tube	2447 reflections with *I* > 2σ(*I*)
graphite	*R*_int_ = 0.027
φ and ω scans	θ_max_ = 27.0°, θ_min_ = 2.3°
Absorption correction: multi-scan (*SADABS*; Sheldrick, 1996)	*h* = −14→14
*T*_min_ = 0.884, *T*_max_ = 0.919	*k* = −19→19
11365 measured reflections	*l* = −10→10

### Refinement

**Table:**

Refinement on *F*^2^	Primary atom site location: structure-invariant direct methods
Least-squares matrix: full	Secondary atom site location: difference Fourier map
*R*[*F*^2^ > 2σ(*F*^2^)] = 0.030	Hydrogen site location: inferred from neighbouring sites
*wR*(*F*^2^) = 0.080	H-atom parameters constrained
*S* = 1.04	*w* = 1/[σ^2^(*F*_o_^2^) + (0.0375*P*)^2^ + 0.7004*P*] where *P* = (*F*_o_^2^ + 2*F*_c_^2^)/3
2768 reflections	(Δ/σ)_max_ = 0.001
156 parameters	Δρ_max_ = 0.38 e Å^−^^3^
0 restraints	Δρ_min_ = −0.43 e Å^−^^3^

### Special details

**Table:**

Geometry. All e.s.d.'s (except the e.s.d. in the dihedral angle between two l.s. planes) are estimated using the full covariance matrix. The cell e.s.d.'s are taken into account individually in the estimation of e.s.d.'s in distances, angles and torsion angles; correlations between e.s.d.'s in cell parameters are only used when they are defined by crystal symmetry. An approximate (isotropic) treatment of cell e.s.d.'s is used for estimating e.s.d.'s involving l.s. planes.
Refinement. Refinement of *F*^2^ against ALL reflections. The weighted *R*-factor *wR* and goodness of fit *S* are based on *F*^2^, conventional *R*-factors *R* are based on *F*, with *F* set to zero for negative *F*^2^. The threshold expression of *F*^2^ > σ(*F*^2^) is used only for calculating *R*-factors(gt) *etc*. and is not relevant to the choice of reflections for refinement. *R*-factors based on *F*^2^ are statistically about twice as large as those based on *F*, and *R*- factors based on ALL data will be even larger.

### Fractional atomic coordinates and isotropic or equivalent isotropic displacement parameters (Å^2^)

**Table:**

	*x*	*y*	*z*	*U*_iso_*/*U*_eq_	
Cl1	1.15452 (4)	0.78261 (3)	0.78766 (6)	0.03679 (13)	
S1	0.67185 (3)	0.54702 (2)	0.90462 (5)	0.01583 (10)	
O1	0.57169 (10)	0.60996 (7)	0.84129 (15)	0.0224 (2)	
O2	0.76889 (10)	0.56910 (7)	1.06785 (14)	0.0236 (2)	
N1	0.73994 (11)	0.53279 (8)	0.74623 (16)	0.0169 (3)	
C1	0.72390 (16)	0.49244 (12)	0.4367 (2)	0.0266 (4)	
H1A	0.6744	0.4541	0.3397	0.040*	
H1B	0.8133	0.4784	0.4645	0.040*	
H1C	0.7095	0.5542	0.3992	0.040*	
C2	0.68489 (13)	0.47796 (10)	0.59948 (19)	0.0180 (3)	
C3	0.60397 (14)	0.41358 (10)	0.6087 (2)	0.0195 (3)	
H3	0.5626	0.3814	0.5029	0.023*	
C4	0.57720 (13)	0.39124 (9)	0.7697 (2)	0.0181 (3)	
C5	0.51384 (16)	0.30477 (10)	0.7770 (2)	0.0264 (3)	
H5A	0.4946	0.3006	0.8898	0.040*	
H5B	0.5697	0.2563	0.7697	0.040*	
H5C	0.4358	0.3010	0.6763	0.040*	
C6	0.61295 (13)	0.44386 (9)	0.9154 (2)	0.0171 (3)	
H6	0.6053	0.4233	1.0254	0.021*	
C7	0.84013 (13)	0.59335 (10)	0.75099 (19)	0.0173 (3)	
C8	0.81380 (15)	0.68225 (10)	0.7151 (2)	0.0233 (3)	
H8	0.7297	0.7028	0.6841	0.028*	
C9	0.91055 (15)	0.74081 (11)	0.7247 (2)	0.0270 (4)	
H9	0.8936	0.8017	0.7006	0.032*	
C10	1.03192 (15)	0.70909 (11)	0.7698 (2)	0.0243 (3)	
C11	1.05906 (14)	0.62056 (11)	0.8042 (2)	0.0237 (3)	
H11	1.1431	0.6000	0.8337	0.028*	
C12	0.96219 (14)	0.56223 (10)	0.7952 (2)	0.0202 (3)	
H12	0.9794	0.5013	0.8191	0.024*	

### Atomic displacement parameters (Å^2^)

**Table:**

	*U*^11^	*U*^22^	*U*^33^	*U*^12^	*U*^13^	*U*^23^
Cl1	0.0280 (2)	0.0363 (2)	0.0456 (3)	−0.01448 (18)	0.01096 (19)	0.00453 (19)
S1	0.01745 (18)	0.01486 (18)	0.01607 (18)	−0.00151 (13)	0.00658 (14)	−0.00116 (13)
O1	0.0240 (6)	0.0180 (5)	0.0280 (6)	0.0041 (4)	0.0121 (5)	0.0018 (4)
O2	0.0258 (6)	0.0265 (6)	0.0175 (5)	−0.0090 (5)	0.0051 (5)	−0.0038 (4)
N1	0.0166 (6)	0.0177 (6)	0.0175 (6)	−0.0017 (5)	0.0071 (5)	−0.0011 (5)
C1	0.0287 (8)	0.0336 (9)	0.0188 (8)	−0.0013 (7)	0.0094 (7)	−0.0017 (6)
C2	0.0170 (7)	0.0204 (7)	0.0157 (7)	0.0049 (6)	0.0038 (6)	0.0001 (6)
C3	0.0188 (7)	0.0191 (7)	0.0184 (7)	0.0016 (6)	0.0028 (6)	−0.0035 (6)
C4	0.0151 (7)	0.0156 (7)	0.0224 (7)	0.0017 (5)	0.0045 (6)	0.0007 (6)
C5	0.0303 (8)	0.0179 (7)	0.0306 (8)	−0.0065 (7)	0.0093 (7)	−0.0030 (6)
C6	0.0172 (7)	0.0154 (7)	0.0196 (7)	−0.0010 (5)	0.0070 (6)	0.0022 (5)
C7	0.0164 (7)	0.0201 (7)	0.0163 (7)	−0.0009 (6)	0.0062 (5)	0.0019 (5)
C8	0.0181 (7)	0.0217 (8)	0.0298 (8)	0.0025 (6)	0.0070 (6)	0.0053 (6)
C9	0.0271 (8)	0.0193 (8)	0.0349 (9)	0.0000 (7)	0.0103 (7)	0.0063 (7)
C10	0.0204 (8)	0.0278 (8)	0.0249 (8)	−0.0079 (6)	0.0077 (6)	0.0028 (6)
C11	0.0164 (7)	0.0296 (8)	0.0252 (8)	0.0021 (6)	0.0069 (6)	0.0048 (6)
C12	0.0206 (7)	0.0206 (7)	0.0199 (7)	0.0019 (6)	0.0072 (6)	0.0034 (6)

### Geometric parameters (Å, °)

**Table:**

Cl1—C10	1.7416 (16)	C4—C5	1.501 (2)
S1—O2	1.4369 (11)	C5—H5A	0.9800
S1—O1	1.4382 (11)	C5—H5B	0.9800
S1—N1	1.6703 (12)	C5—H5C	0.9800
S1—C6	1.7104 (14)	C6—H6	0.9500
N1—C2	1.3987 (19)	C7—C12	1.385 (2)
N1—C7	1.4430 (18)	C7—C8	1.390 (2)
C1—C2	1.496 (2)	C8—C9	1.386 (2)
C1—H1A	0.9800	C8—H8	0.9500
C1—H1B	0.9800	C9—C10	1.381 (2)
C1—H1C	0.9800	C9—H9	0.9500
C2—C3	1.351 (2)	C10—C11	1.384 (2)
C3—C4	1.433 (2)	C11—C12	1.386 (2)
C3—H3	0.9500	C11—H11	0.9500
C4—C6	1.350 (2)	C12—H12	0.9500
			
O2—S1—O1	116.33 (7)	H5A—C5—H5B	109.5
O2—S1—N1	107.56 (6)	C4—C5—H5C	109.5
O1—S1—N1	108.60 (6)	H5A—C5—H5C	109.5
O2—S1—C6	111.35 (7)	H5B—C5—H5C	109.5
O1—S1—C6	110.61 (7)	C4—C6—S1	120.91 (12)
N1—S1—C6	101.21 (7)	C4—C6—H6	119.5
C2—N1—C7	122.44 (12)	S1—C6—H6	119.5
C2—N1—S1	120.46 (10)	C12—C7—C8	120.75 (14)
C7—N1—S1	115.81 (10)	C12—C7—N1	119.36 (13)
C2—C1—H1A	109.5	C8—C7—N1	119.87 (13)
C2—C1—H1B	109.5	C9—C8—C7	119.85 (14)
H1A—C1—H1B	109.5	C9—C8—H8	120.1
C2—C1—H1C	109.5	C7—C8—H8	120.1
H1A—C1—H1C	109.5	C10—C9—C8	118.88 (15)
H1B—C1—H1C	109.5	C10—C9—H9	120.6
C3—C2—N1	120.91 (13)	C8—C9—H9	120.6
C3—C2—C1	122.43 (14)	C9—C10—C11	121.75 (15)
N1—C2—C1	116.63 (13)	C9—C10—Cl1	119.19 (13)
C2—C3—C4	123.45 (14)	C11—C10—Cl1	119.05 (12)
C2—C3—H3	118.3	C10—C11—C12	119.23 (14)
C4—C3—H3	118.3	C10—C11—H11	120.4
C6—C4—C3	121.54 (13)	C12—C11—H11	120.4
C6—C4—C5	120.04 (14)	C7—C12—C11	119.54 (14)
C3—C4—C5	118.34 (13)	C7—C12—H12	120.2
C4—C5—H5A	109.5	C11—C12—H12	120.2
C4—C5—H5B	109.5		

### Hydrogen-bond geometry (Å, °)

**Table:**

*D*—H···*A*	*D*—H	H···*A*	*D*···*A*	*D*—H···*A*
C6—H6···O1^i^	0.95	2.57	3.3384 (18)	138.
C9—H9···O2^ii^	0.95	2.44	3.3339 (19)	156.

Symmetry codes: (i) −*x*+1, −*y*+1, −*z*+2; (ii) *x*, −*y*+3/2, *z*−1/2.
